# Quetiapine Moderates Doxorubicin-Induced Cognitive Deficits: Influence of Oxidative Stress, Neuroinflammation, and Cellular Apoptosis

**DOI:** 10.3390/ijms241411525

**Published:** 2023-07-16

**Authors:** Vasudevan Mani, Bander Shehail Alshammeri

**Affiliations:** Department of Pharmacology and Toxicology, College of Pharmacy, Qassim University, Buraydah 51452, Saudi Arabia

**Keywords:** chemotherapy-induced cognitive deficits, atypical antipsychotic drugs, oxidative stress, neuroinflammation, apoptosis

## Abstract

Chemotherapy is considered a major choice in cancer treatment. Unfortunately, several cognitive deficiencies and psychiatric complications have been reported in patients with cancer during treatment and for the rest of their lives. Doxorubicin (DOX) plays an important role in chemotherapy regimens but affects both the central and peripheral nervous systems. Antipsychotic drugs alleviate the behavioral symptoms of aging-related dementia, and the atypical class, quetiapine (QUET), has been shown to have beneficial effects on various cognitive impairments. The present investigation aimed to determine the possible mechanism underlying the effect of thirty-day administrations of QUET (10 or 20 mg/kg, p.o.) on DOX-induced cognitive deficits (DICDs). DICDs were achieved through four doses of DOX (2 mg/kg, i.p.) at an interval of seven days during drug treatment. Elevated plus maze (EPM), novel object recognition (NOR), and Y-maze tasks were performed to confirm the DICDs and find the impact of QUET on them. The ELISA tests were executed with oxidative [malondialdehyde (MDA), catalase, and reduced glutathione (GSH)], inflammatory [cyclooxygenase-2 (COX-2), nuclear factor kappa B (NF-κB), and tumor necrosis factor-alpha (TNF-α)], and apoptosis [B-cell lymphoma 2 (Bcl2), Bcl2 associated X protein (Bax), and Caspase-3] markers were assessed in the brain homogenate to explore the related mechanisms. DICD lengthened the transfer latency time in EPM, shortened the exploration time of the novel object, reduced the discrimination ability of the objects in NOR, and lowered the number of arm entries and time spent in the novel arm. QUET alleviated DICD-related symptoms. In addition, QUET reduced neuronal oxidative stress by reducing MDA and elevating GSH levels in the rat brain. Moreover, it reduced neuronal inflammation by controlling the levels of COX-2, NF-κB, and TNF-α. By improving the Bcl-2 level and reducing both Bax and Caspase-3 levels, it protected against neuronal apoptosis. Collectively, our results supported that QUET may protect against DICD, which could be explained by the inhibition of neuronal inflammation and the attenuation of cellular apoptosis protecting against oxidative stress.

## 1. Introduction

Cognitive deficits are a documented clinical phenomenon that have been connected to chemotherapy-related cancer survival. Between 13 and 78% of patients undergoing chemotherapy have been found to have neurological-related complications, which significantly affect the therapeutic outcomes of their treatment [1]. Several symptoms, including memory loss, slow processing speed, trouble focusing, and language issues, have been reported by cancer survivors. Cognitive decline with chemotherapy is termed “chemo-fog” or “chemo-brain” and is majorly characterized by “fuzzy-headiness” or “mental slowness” [2]. Moreover, chemotherapy has been linked to a number of psychiatric side effects, such as psychosis, anxiety, and mania. According to a review, a list of secondary psychological symptoms such as being overly talkative, irritable mood, insomnia, high self-esteem, and hyperreligiosity have been reported in some patients after chemotherapy treatments [3]. Crosstalk exists between psychotic symptoms and the experience of cognitive impairment. Moreover, most schizophrenia patients demonstrate cognitive decline from moderate to severe levels when linked to healthy samples [4]. However, the specific mechanisms underlying cognitive deficits induced by chemotherapy drugs are uncertain. Reports have listed some related mechanisms, such as direct neuronal toxicity, immune modifications, the elevation of inflammatory mediators, cellular apoptosis process, and oxidative vulnerability [5,6]. 

The anthracycline antibiotic doxorubicin (DOX) is the most effective therapy for a variety of cancers, particularly solid tumors like breast, lung, ovarian, and gastric cancers, lymphoma, and pediatric leukemia [7]. Principally, DOX exerts its cytotoxic effects through different pathways, among which affecting DNA synthesis by interfering with topoisomerase II is essential. Unexpectedly, like most chemotherapy agents, DOX therapeutic utilization is also limited by its extended cytotoxicity, as reported for a range of organs such as the heart, liver, and brain [8]. Although DOX has limitations in the penetration of the blood–brain barrier (BBB) due to multidrug resistance efflux transporters and its ionization nature in peripheral circulation, it can still result in extensive toxicity of the brain tissues. In the periphery, DOX treatment has been shown to result in oxidative stress by liberating reactive oxygen species (ROS), which induce the peripheral proinflammatory cytokine tumor necrosis factor-alpha (TNF-α). It can penetrate the BBB and cause neuronal toxicity by the further local production of TNF-α and other proinflammatory cytokines like interleukin (IL)-6 and IL-1β [8,9,10]. However, recent evidence supports the direct neuronal toxicity of DOX via crossing the BBB. Studies have shown that the presence of DOX in the brain is followed by peripheral administration [11,12,13]. In addition, communication between neuronal stem cells (NSCs) and blood vessels has been explained recently, and it has been reported that DOX is selectively and readily taken up by hippocampal NSCs through vascular-associated apical projections [14]. Du et al. reported that DNA damage, the interruption of the normal cell cycle, the elevation of oxidative stress, mitochondrial dysfunctions, the activation of apoptosis pathways, interference in neurogenesis, the modulation of neurotransmitters, abnormal brain synaptic plasticity, and alteration of protein kinase pathways are related mechanisms of DOX-induced direct neurotoxicity [11]. 

Quetiapine (QUET), a novel atypical antipsychotic medication, successfully reduces schizophrenia patients’ positive and negative symptoms as well as their cognitive impairment. The antipsychotic mechanism of QUET is related to the potential inhibition of the serotonin 5HT_2A_ receptor and its lower affinity to the dopamine D_2_ receptor, which differs from typical antipsychotics [15]. In schizophrenia with negative symptoms, QUET therapy improved patients’ cognitive index scores at weeks 6 and 12, and the extent of the study revealed that the level of cognitive performance was higher than that of other drugs from the same classifications, such as aripiprazole, risperidone, and olanzapine [16,17]. In animal models, using an amyloid precursor protein (APP)/presenilin-1 (PS-1) double transgenic (TG) mouse model of AD, treatment with QUET attenuated memory impairment, decreased the number of β-amyloid (Aβ) plaques and up-regulated the cerebral anti-apoptosis B-cell lymphoma (Bcl)-2 protein [18]. Additionally, improvements in recognition memory and protection from hippocampal oxidative stress by reducing nitrotyrosine have also been reported in APP/PS-1 TG mice [19]. Furthermore, treatment with QUET ameliorated phencyclidine-induced reference memory deficits and the progression of brain apoptosis by decreasing the Bcl-XL/Bcl2 associated X protein (Bax) ratio [20]. Additionally, it reversed methamphetamine-induced recognition memory impairment and dopaminergic neuronal deficits in the brain striatum [21]. Recently, QUET treatment was shown to inhibit neuroinflammation in different animal models by altering inflammatory mediators. In diabetes-induced mice, it controlled the release of inflammatory cytokines by inhibiting the activation of brain astrocytes and microglial cells [22]. Also, QUET and its metabolite norquetiapine were evidenced to ameliorate lipopolysaccharide (LPS)-induced hippocampal inflammation in mice [15]. Collectively, we hypothesized that QUET could alleviate DOX-induced neuronal damage in the brain due to inflammation, cellular apoptosis, and oxidative vulnerability. To test our hypothesis, the current research was designed to investigate the benefits of QUET on DOX-induced neurotoxicity, including cognitive impairments, oxidative vulnerability, neuronal inflammation, and apoptosis process in rats.

## 2. Results

### 2.1. QUET-Attenuated Memory Deficits in DOX-Induced Rats in an Elevated Plus Maze (EPM) Test

**Figure 1:**
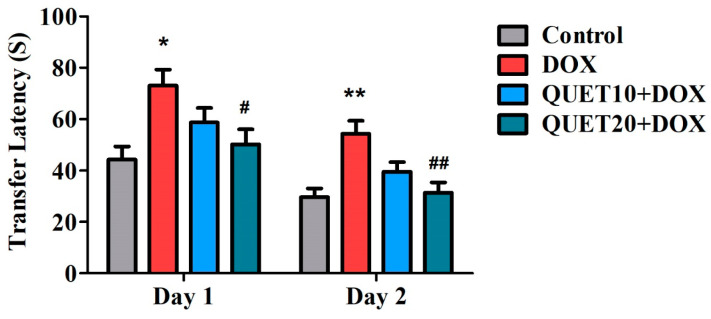
Quetiapine (QUET) reduced doxorubicin (DOX)-induced transfer latency in the elevated plus maze test. The results are expressed by mean ± SEM (*n* = 6). One-way ANOVA [F(3,20) = 4.689, *p* < 0.05 for day 1 and F(3,20) = 7.560, *p* < 0.01 for day 2] followed by Tukey–Kramer multiple comparisons tests were conducted: * *p* < 0.05 and ** *p* < 0.01 as compared to the control group; # *p* < 0.05 and ## *p* < 0.01 as compared to the DOX-induced group.

The EPM test assessed the transfer latency (TL) time of the treated rats. Shorter TL values indicated the facilitation of their learning as well as memory capability. The effects of QUET and DOX treatments on day 1 and day 2 TL values are shown in Figure 1. One-way ANOVA indicated variations in TL performance among the groups on day 1 [F(3,20) = 4.689, *p* < 0.05] and on day 2 [F(3,20) = 7.560, *p* < 0.01], which highlighted the alteration of QUET on memory functions. Furthermore, on day 1 of testing, the group of rats treated with four doses of DOX injection showed a prolonged TL time (73.00 ± 6.26 s; *p* < 0.05) as compared to the control group (44.33 ± 5.03 s), representing a decline in rats’ learning capability after DOX administration. In addition, QUET at 20 mg/kg treatment resulted in a shorter TL time (50.17 ± 5.902 s; *p* < 0.05) with DOX induction, which supported the alteration of QUET’s learning ability in rats. In parallel, on day 2, the extension of TL time (54.33 ± 5.04 s; *p* < 0.01) of the DOX-induced group as compared to the control animals (29.67 ± 3.34 s) further confirmed the DOX-induced memory deficits. Continuous treatment with QUET (20 mg/kg, p.o.) could protect against such deficits by lowering the TL time (31.33 ± 4.01 s) and improving maze performance.

### 2.2. QUET-Improved Cognitive Functions in DOX-Induced Rats in Novel Object Recognition (NOR) Test

**Figure 2:**
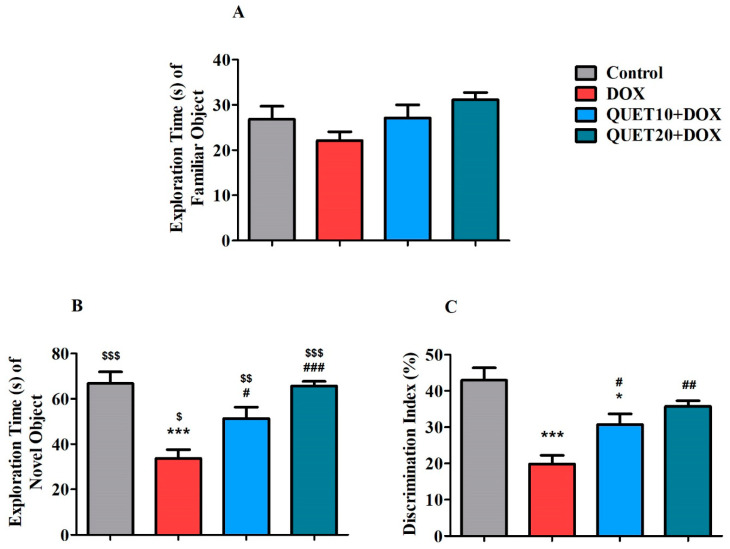
Impact of quetiapine (QUET) on doxorubicin (DOX)-induced (**A**) exploration time of a familiar object (FO), (**B**) exploration time of a novel object (NO), and (**C**) discrimination index (DI) during the test session in novel object recognition test. The results are expressed by mean ± SEM (*n* = 6). One-way ANOVA [F(3,20) = 2.402, *p* > 0.05 for exploration time of FO; F(3,20) = 13.84, *p* < 0.001 for exploration time of NO; F(3,20) = 13.59, *p* < 0.001 for DI] followed by Tukey–Kramer multiple comparisons tests were conducted for comparisons between the groups: * *p* < 0.05 and *** *p* < 0.001 as compared to the control group; # *p* < 0.05, ## *p* < 0.01, and ### *p* < 0.001 as compared to the DOX-induced group; $ *p* < 0.05, $$ *p* < 0.01, and $$$ *p* < 0.001 as compared to the corresponding groups of FO1 and NO.

The NOR test was used to assess the rats’ recognition memory following QUET and DOX therapies (Figure 2). During the test session, each rat was allowed to explore both familiar (FO) and novel (NO) objects. Further, the exploration time of FO as well as NO, and also the percentage of discrimination index (PDI), was calculated to identify animals’ discrimination capability between the objects. Concerning the exploration time of FO, there was no considerable alteration from either QUET or DOX treatments (Figure 2A). In addition, considering the exploration time of NO, both treatments altered the exploration time [F(3,20) = 13.84, *p* < 0.001] between the treated groups (Figure 2B). Following the post hoc analysis between the two groups, there was a significant variation (*p* < 0.001) in the exploration time of NO between the control (66.83 ± 5.06 s) and DOX-induction (33.67 ± 3.89 s) groups. The decrease in exploration time following DOX administration could explain the DICD in rats. Notably, the concurrent administration of QUET with 10 mg/kg (51.33 ± 4.98 s; *p* < 0.05) and 20 mg/kg (65.67 ± 1.98 s; *p* < 0.001) prolonged the exploration of NO in DOX-induced rats. The evaluation of exploration time between the objects (FO1 and NO) using an unpaired t-test clarified that rats from each group spent more time with NO than with FO1 (control: *p* < 0.001, DOX: *p* < 0.05, QUET10 + DOX: *p* < 0.01, and QUET20 + DOX: *p* < 0.001).

Further, as shown in Figure 2C, one-way ANOVA analysis highlighted that treatment with QUET and DOX produced significant changes [F(3,20) = 13.59, *p* < 0.001] in PDI among the groups. Similarly, the DICD was reflected by lowering the PDI in DOX-induced rats (19.85 ± 2.38%, *p* < 0.001) in comparison with the control (42.98 ± 3.39%). In addition, increasing the PDI through QUET treatment (30.78 ± 2.86%, *p* < 0.05 for 10 mg/kg and 35.74 ± 1.58%, *p* < 0.01 for 20 mg/kg) in DOX-induced rats supported the reversal of discrimination ability in QUET-treated rats.

### 2.3. QUET-Enhanced Cognitive Functions in DOX-Induced Rats in Y-Maze Test

**Figure 3:**
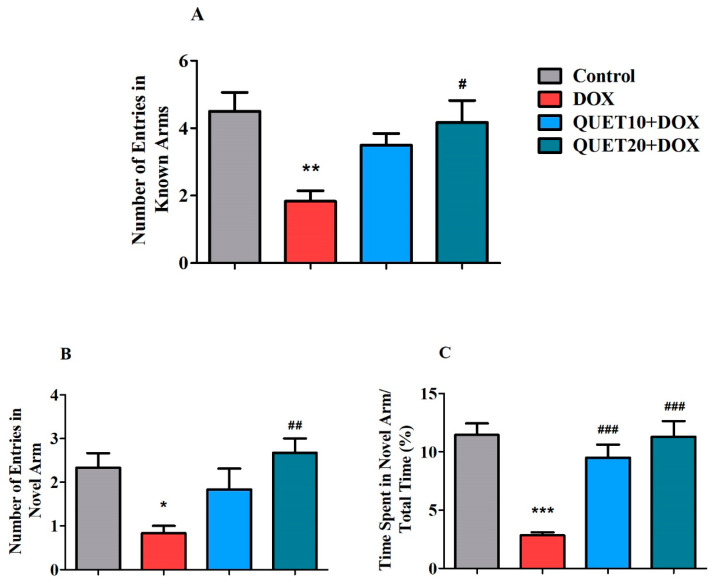
Quetiapine (QUET) alters doxorubicin (DOX)-induced (**A**) number of entries in known arms, (**B**) number of entries in the novel arm, and (**C**) percentage of time spent in the novel arm during the test session in Y-maze. The results are expressed by mean ± SEM (*n* = 6). One-way ANOVA [F(3,20) = 5.891, *p* < 0.01 for the number of entries in the known arm; F(3,20) = 5.349, *p* < 0.01 for the number of entries in a novel arm; F(3,20) = 16.00, *p* < 0.001 for the percentage of time spent in the novel arm] followed by Tukey–Kramer multiple comparisons tests were conducted: * *p* < 0.05, ** *p* < 0.01, and *** *p* < 0.001 as compared to the control group; # *p* < 0.05, ## *p* < 0.01, and ### *p* < 0.001 as compared to the DOX-induced group.

Figure 3 shows the effect of QUET and DOX on the modification of rat behaviors, namely novel arm exploration and adaptation to a new environment, which were studied in the Y-maze test. Here, the number of known and novel arm entries, and the percentage of time spent in the novel arm, were analyzed during a test session. A one-way ANOVA revealed a significant impact of QUET and DOX on the number of entries into the known arms [F(3,20) = 5.891, *p* < 0.01], the novel arm [F(3,20) = 5.349, *p* < 0.01], and the percentage of time spent in the novel arm [F(3,20) = 16.00, *p* < 0.001]. Specifically, DOX administration showed its deficits by reducing the number of known arm entries (1.833 ± 0.31, *p* < 0.01) compared to control rats (4.500 ± 5.56; *p* < 0.05). In addition, a higher dose of QUET (20 mg/kg, p.o.) increased the number of entries (4.167 ± 0.65; *p* < 0.05) successfully, as observed in DOX-induced rats (Figure 3A). 

The number of entries into the novel arm was also affected by DOX administration (0.833 ± 0.17; *p* < 0.05) compared to that in the control group performance (2.333 ± 0.33) (Figure 3B). Interestingly, QUET (20 mg/kg) increased the number of novel arm entries (2.667 ± 0.33; *p* < 0.01). Likewise, DOX induction led to a shorter percentage of time spent by animals in the novel arm (2.833 ± 0.25%, *p* < 0.001) in comparison with the control results (11.45 ± 0.98%). The DICDs were counteracted by QUET treatment, resulting in an increased percentage of time spent in the novel arm (9.500 ± 1.10%, *p* < 0.001 for 10 mg/kg and 11.28 ± 1.36%, *p* < 0.001 for 20 mg/kg) (Figure 3C).

### 2.4. QUET-Ameliorated Oxidative Stress in Brain Tissues of DOX-Induced Rats

**Figure 4:**
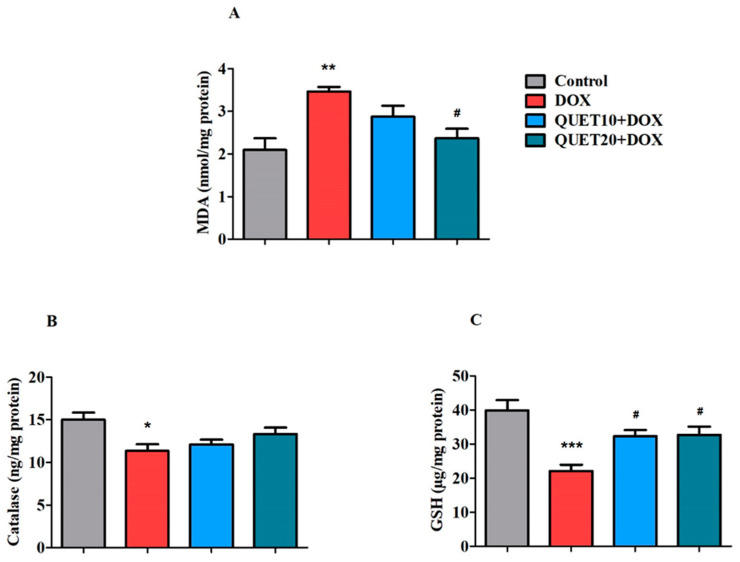
Quetiapine (QUET) reduced doxorubicin (DOX)-induced oxidative stress by modifying the levels of (**A**) malondialdehyde (MDA), (**B**) catalase, and (**C**) reduced glutathione (GSH) in the rats’ brains. The results are expressed by mean ± SEM (*n* = 6). One-way ANOVA [F(3,20) = 7.183, *p* < 0.01 for MDA; F(3,20) = 4.598, *p* < 0.05 for catalase; and F(3,20) = 9.929, *p* < 0.001 for GSH] followed by Tukey–Kramer multiple comparisons tests were conducted for comparisons between the groups: * *p* < 0.05, ** *p* < 0.01, and *** *p* < 0.001 as compared to the control group; # *p* < 0.05 as compared to the DOX-induced group.

The impact of QUET on DOX-induced oxidative damage was further examined by measuring oxidative and antioxidant indicators such as malondialdehyde (MDA), catalase, and reduced glutathione (GSH) in brain homogenates (Figure 4). 

MDA is a key end product of lipid peroxidation and an indicator of oxidative stress. Among the treatment groups, MDA levels in the brain were significantly changed [F(3,20) = 7.183, *p* < 0.01] by QUET and DOX administration (Figure 4A). Four doses of DOX induction resulted in oxidative damage to the brain, indicated by the elevation of MDA levels (3.466 ± 0.109 nmol/mg protein, *p* < 0.01), which matched the control levels (2.094 ± 0.269 nmol/mg protein). The results found that QUET at 20 mg/kg could moderately decline oxidative damage by reducing brain MDA levels (2.365 ± 0.228 nmol/mg protein) in DOX-induced rats. 

The antioxidant enzyme catalase level was affected by DOX administration [F(3,20) = 4.598, *p* < 0.05] but was not modified by QUET treatment in rat brains (Figure 4B). The level of catalase in DOX-induced animals was 11.36 ± 0.764 ng/mg protein (*p* < 0.05), compared to the control animals’ level of 15.01 ± 0.8370 ng/mg protein. 

The levels of another antioxidant marker, GSH, were significantly modified [F(3,20) = 9.929, *p* < 0.001] after the successful administration of QUET and DOX in the brain when one-way ANOVA analysis was performed between all treated groups (Figure 4C). Furthermore, in comparison with the control group (39.91 ± 2.994 µg/mg protein), it was estimated that DOX administration showed a significant decline in brain GSH levels (22.11 ± 1.817 µg/mg protein, *p* < 0.001). Nevertheless, subsequent treatment with QUET improved the antioxidant capability by significantly elevating (*p* < 0.05) the GSH levels (32.22 ± 1.789 µg/mg protein with 10 mg/kg and 32.67 ± 2.471 µg/mg protein with 20 mg/kg) in the brain homogenate.

### 2.5. QUET-Reduced Neuro-Inflammatory Mediators in the Brain Tissues of DOX-Induced Rats

**Figure 5:**
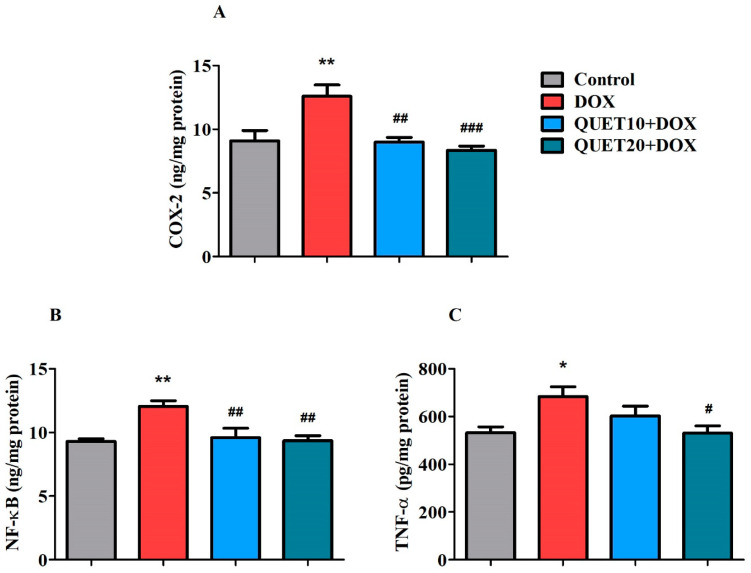
Quetiapine (QUET) suppressed doxorubicin (DOX)-induced neuroinflammation by reducing the levels of (**A**) cyclooxygenase-2 (COX-2), (**B**) nuclear factor kappa B (NF-κB), and (**C**) tumor necrosis factor-alpha (TNF-α) in the rats’ brains. The results are expressed by mean ± SEM (*n* = 6). One-way ANOVA [F(3,20) = 8.538, *p* < 0.001 for COX-2; F(3,20) = 7.683, *p* < 0.01 for NF-κB; F(3,20) = 4.483, and *p* < 0.05 for TNF-α] followed by Tukey–Kramer multiple comparison tests were conducted for comparison between the groups: * *p* < 0.05 and ** *p* < 0.01 as compared to the control group; # *p* < 0.05, ## *p* < 0.01, and ### *p* < 0.001 as compared to DOX-induced group.

Three selective inflammatory markers—cyclooxygenase-2 (COX-2), nuclear factor kappa B (NF-κB), and TNF-α—were assessed in brain tissues to determine whether QUET could modify DOX-induced neuronal inflammatory insults (Figure 5). 

It was found that there was a remarkable difference in the levels of COX-2 [F(3,20) = 8.538, *p* < 0.001], an inflammation-inducible enzyme, in the brains of all the treated groups (Figure 5A). After the four doses of DOX injection, the level of the brain COX-2 (12.58 ± 0.90 ng/mg protein, *p* < 0.01) was increased when compared to control levels (9.093 ± 0.81 ng/mg protein). However, QUET treatment with 10 mg/kg (8.995 ± 0.36 ng/mg protein, *p* < 0.01) and 20 mg/kg (8.360 ± 0.32 ng/mg protein, *p* < 0.001) reduced the DOX-induced COX-2 levels in brain tissues.

The protein transcription factor NF-κB was measured in brain homogenates. Significant effects [F(3,20) = 7.683, *p* < 0.01] were observed in its levels among all the treatment groups (Figure 5B) when using one-way ANOVA. The level of NF-κB was significantly raised (12.06 ± 0.43 ng/mg protein, *p* < 0.01) by DOX when compared to the control treatment (9.298 ± 0.23 ng/mg protein). Parallel administration of QUET with both doses (9.591 ± 0.74 ng/mg protein, *p* < 0.01 at 10 mg/kg and 9.360 ± 0.37 ng/mg protein, *p* < 0.01 at 20 mg/kg) significantly controlled the elevated levels of NF-κB in DOX-induced rats. 

Additionally, the levels of one of the key proinflammatory cytokines, TNF-α, were also analyzed in the brain homogenates from all treatments. Evidently, the TNF-α levels were moderately altered [F(3,20) = 4.483, *p* < 0.05] following the treatment with QUET and DOX (Figure 5C). As expected, the TNF-α level was elevated (684.3 ± 39.91 pg/mg protein, *p* < 0.05) by DOX induction as compared to the control rats (531.5 ± 24.62 pg/mg protein). Moreover, the elevated level of TNF-α in the brain homogenate was considerably reduced (530.6 ± 29.83 pg/mg protein, *p* < 0.05) by a higher dose (20 mg/kg, p.o.) of QUET treatment. 

### 2.6. QUET-Reduced Apoptosis in the Brain Tissues of DOX-Induced Rats

**Figure 6:**
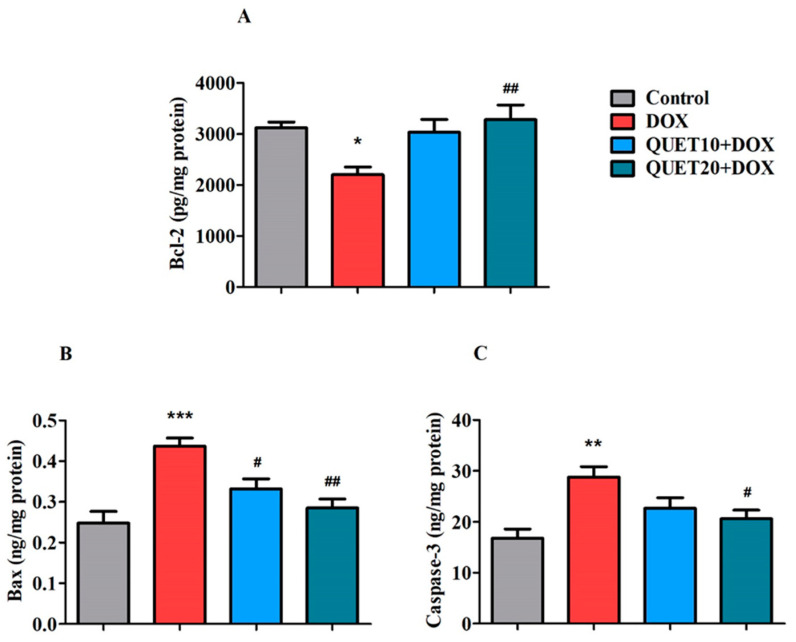
Quetiapine (QUET) reduced doxorubicin (DOX)-induced neuronal apoptosis by altering the levels of (**A**) B-cell lymphoma 2 (Bcl-2), (**B**) Bcl2 associated X protein (Bax), and (**C**) Caspase-3 in the rats’ brains. The results are expressed by mean ± SEM (n = 6). One-way ANOVA [F(3,20) = 5.196, *p* < 0.01 for Bcl-2; F(3,20) = 11.74, *p* < 0.001 for Bax; and F(3,20) = 6.866, *p* < 0.01 for Caspase-3] followed by Tukey–Kramer multiple comparison tests were conducted for comparisons between the groups: * *p* < 0.05, ** *p* < 0.01, and *** *p* < 0.001 as compared to the control group; # *p* < 0.05 and ## *p* < 0.01 as compared to DOX-induced group.

To analyze the effect of QUET on DOX-induced apoptotic damage in brain tissue, the present study aimed to estimate three apoptotic proteins: Bcl2, Bax, and Caspase-3 (Figure 6).

According to the one-way ANOVA analysis, the levels of the anti-apoptotic protein Bcl-2 were significantly altered [F(3,20) = 5.196, *p* < 0.01] following treatment with QUET and DOX (Figure 6A). Compared to the control animals (3124 ± 112.2 pg/mg protein), the DOX-induced group showed a lower level (2204 ± 150.0 pg/mg protein, *p* < 0.05) of Bcl-2 in brain tissues. The QUET (20 mg/kg, p.o.) treatment effectively raised the Bcl-2 levels (3286 ± 284.9 pg/mg protein, *p* < 0.01) as compared to the DOX-induced animals.

As shown in Figure 6B, the levels of pro-apoptotic protein Bax were found to be significantly different between the groups [F(3,20) = 11.74, *p* < 0.001], according to the one-way ANOVA analysis. DOX induction resulted in a higher level of Bax (0.4368 ± 0.019 ng/mg protein, *p* < 0.001) in the brain, as referred to the control treatment (0.2478 ± 0.029 ng/mg protein). However, the elevated Bax levels induced by DOX were significantly reduced after QUET treatments (0.3320 ± 0.024 ng/mg protein, *p* < 0.05 with 10 mg/kg and 0.2847 ± 0.022 ng/mg protein, *p* < 0.05 with 20 mg/kg).

In addition, the levels of the second targeted protein from the pro-apoptosis family, Caspase-3m were analyzed in the rat brains from various treatment groups (Figure 6C), and it was found that the levels of Caspase-3 were significantly altered [F(3,20) = 6.866, *p* < 0.01] by various treatments. The level of Caspase-3 in DOX-induced animals was 28.78 ± 2.037 ng/mg protein, which was higher (*p* < 0.01) than the control treatment level (16.82 ± 1.784 ng/mg protein). The level of Caspase-3 was reduced (*p* < 0.05) to 20.63 ± 1.659 ng/mg protein after treatment with QUET at 20 mg/kg.

## 3. Discussion

DOX-associated cognitive deficiency has drawn much attention because it negatively impacts cancer patients’ quality of life [23]. Meanwhile, several clinical and preclinical behavior-related studies have shown that QUET from the atypical antipsychotic class is an attractive target for memory impairment associated with schizophrenia, Alzheimer’s disease (AD), and drug-induced cognitive deficits [16,17,18,19,20,21]. The results from our present study also support this previous evidence. Parallel administration of QUET attenuated DOX-induced behavioral changes and reduced neuroinflammatory mediators, apoptosis-induced markers, and oxidative vulnerability in a rat model. 

The behavioral changes induced by QUET and DOX have been documented using selective tasks such as EPM, NOR, and Y-Maze. For this experiment, QUET was administered for 30 days at two dose levels (10 or 20 mg/kg, p.o.), and concurrently four doses of DOX (2 mg/kg, i.p.) were injected at an interval of seven days during the entire treatment schedule. Initially, the EPM test was performed on days 26 and 27 of the treatment timeline. This test is a neutral behavioral model that is widely utilized to study rodents’ behavioral and cognitive functions [5,24]. Generally, the animals dislike staying in open and elevated places and prefer to stay in closed and dark areas. Furthermore, shorter TL values support the improvement of their spatial memory [25]. The results of this present study revealed that the extension of TL values on days 1 and 2 was recorded after four doses of DOX in the EPM test, which indicates impairment of learning and memory in rats. Continuous administration of QUET (20 mg/kg, p.o.) shortened TL values in rats—which had been extended by DOX—on both days. These results suggested that a higher dose of QUET successfully reversed the memory deficits induced by DOX in rats. Consistent with our results, QUET improved anxiety and memory-related behaviors of stressed rats in the EPM test [26]. 

Principally, the NOR test parameters highlight the ability of animals to discriminate between NO and FO, which mainly reflects short-term recognition–working memory. In the second trial with NO and FO, if the rats spent more time exploring the NO, it represented memory for the FO, while if they spent the same amount of time exploring both objects, it indicated the loss of recall or memory for the FO shown during the first trial [27,28]. Interestingly, in the test session (second trial) of the NOR test, both doses of QUET (10 and 20 mg/kg, p.o.) diminished DOX-induced impairment of cognitive parameters, such as exploration time of NO and PDI, when the animals were allowed to explore FO1 and NO. Additionally, the comparison between the exploration time between NO and FO clearly indicated that animals spent more time with NO; in particular, the control and QUET (20 mg/kg, p.o.) treatment groups resulted in significantly higher levels. Consistent with our results, using object recognition tasks, 28 days of QUET administration reversed the methamphetamine-induced decline in exploratory preference of the objects during the retention session performed at 1 h and 24 h after the training session and supported the enhancement of recognition memory in dopaminergic-induced neurotoxicity in rats [21]. Furthermore, in a TG-AD mouse model, prolonged treatment of QUET with drinking water improved the performance of time spent exploring a novel object and improved object recognition memory using object recognition tasks [19]. 

The Y-maze is a behavioral task used to analyze the willingness of rodents to explore a novel environment that reflects their short-term spatial working memory [29]. Rodents often choose to explore a fresh arm of the maze rather than return to one that they have already visited. According to other findings, it is believed that the majority of the brain’s areas, including the hippocampus, prefrontal cortex, septum, and basal forebrain, are involved in this task [29,30]. Our results from the Y-maze test allowed us to investigate the impact of DOX and QUET administrations on different cognitive parameters, such as the number of entries in the known and novel arms, and the percentage of time spent in the novel arm. The DOX induction resulted in a lower number of both known and novel arm entries, and the rats spent less time in the targeted novel arm, which explains the working memory deficits with DOX. Remarkably, treatment with QUET at 20 mg/kg reversed the number of entries into both arms and facilitated higher time spent in the novel arm, indicating attenuation of DOX-induced spatial memory impairment in rats. Previously, cuprizone-induced demyelination mice treated with QUET showed evidence of oligodendrocyte formation and remyelination promotion at weeks 3 and 4, which resulted in the improvement of spatial working memory in the Y-maze test by increasing the animals’ arm alternative behaviors [31]. Further, in a double TG mouse model of AD, QUET enhanced Y-maze performance and cerebral brain-derived neurotrophic factor (BDNF) expression [32]. Correspondingly, QUET protected against acute restraint stress-induced decrease in spontaneous alternation behavior in the Y-maze task and elevation of dopamine D2 receptor expression in the basal ganglia [33]. 

The elevation of oxidative stress causes DOX-induced cognitive impairment through direct and indirect mechanisms [11]. In terms of the chemical structure, the presence of an active redox-quinone moiety is primarily involved in DOX-induced oxidative insults. Specifically, the number of NAD(P)H-dependent oxidoreductase enzymes contributes to DOX metabolism and they liberate harmful superoxide radicals, which are the key precursor for ROS/RNS production [6]. Previous reports supported that DOX can cause direct neurotoxicity by facilitating ROS production and mitochondrial membrane depolarization in neurons [11,34]. In contrast, superoxide from the DOX-quinone moiety leads to higher production of ROS, which elevates the levels of the peripheral inflammatory cytokine TNF-α. Circulatory TNF-α crosses the BBB and leads to further oxidative stress in brain tissue [35]. Regarding oxidative vulnerability, lipids are more targeted and elevate the lipid peroxidation (LPO) levels in tissues. Because of their high content of polyunsaturated fatty acids, brain tissues are more susceptible to oxidative free radicals [36]. Further, MDA is a byproduct of polyunsaturated fatty acid peroxidation and is considered a key marker of LOP [37]. Furthermore, four doses of peripheral DOX injections led to an increase in MDA levels in the brain tissues in the current data, which were also validated by other studies [5,38]. By reducing MDA levels, QUET (20 mg/kg, p.o.) was still efficient in reducing DOX-induced oxidative stress in brain tissues. 

In another way, the antioxidant mechanisms, in particular GSH, act as a defense against the oxidative damages induced by DOX in the brain [6,39]. The critical role of the GSH system has been hypothesized in previous studies [6,40]. In detail, when the DOX-quinone form undergoes metabolism in the NADPH system, it liberates DOX-semiquinone radicals (DOX-SQ•) that can react with free oxygen molecules and produce vulnerable superoxide radicals. Further, superoxide dismutase converts superoxide radicals to hydrogen peroxide, and the presence of catalase converts it to water molecules [6]. In our experimental animals, both doses of QUET treatments failed to alter catalase levels in DOX-induced brain tissues. On the other hand, the GSH system can reduce DOX-SQ• via two different pathways. First, it cross-links with DOX-SQ• and forms a radical addition byproduct called DOX–semiquinone–glutathione adduct. Next, GSH reacts with DOX-SQ• to form a reduced DOX–hydroquinone molecule [6,40]. Interestingly, GSH levels in the DOX-induced brain were elevated by QUET administration at doses of 10 and 20 mg/kg. The elevation of GSH levels by QUET against DOX might be correlated with its oxidative defense system and the reversal of cognitive parameters in rats. In addition, many preclinical models have demonstrated the effectiveness of QUET’s antioxidant properties. In double TG-AD mice, nitrotyrosine, a nitric-oxide-related protein marker of oxidative stress, was altered by QUET treatment to prevent oxidative stress in the hippocampal region [19]. Additionally, both short- and long-term administration of QUET to rats decreased oxidative parameters in the prefrontal cortex (PFC), hippocampus, amygdala, and nucleus accumbens of the brain [41]. The co-administration of QUET also protected against ethanol-induced oxidative vulnerability by decreasing oxidative stress (MDA and ROS levels) and increasing antioxidant capacity (catalase activity and total antioxidant capacity) in the PFC, hippocampus, and cerebellum of rats [42]. 

Increased oxidative insult in the nervous system has also aided in the development of neuroinflammation and the initiation of the apoptotic process in neuronal cells [11]. As discussed earlier, DOX produces a significant amount of ROS in the periphery, which causes more TNF-α to circulate. After passing across the BBB through endocytosis, it stimulates glial cells and the redox-responsive transcription factor NF-κB, which also further increases TNF-α levels as well as other inflammatory mediators, including COX-2, IL-1β, and iNOS in the brain [35,43]. In agreement with this, systemic DOX administration produces inflammatory responses, evidenced by increasing TNF-α and COX-2 levels in the hippocampus and cortex areas [35,44]. Our present results also ‘concurred with previous results: the levels of COX-2, NF-κB, and TNF-α were augmented after four doses of intraperitoneal DOX injections, which revived the neuronal toxicity of DOX. Both doses of QUET (10 and 20 mg/kg, p.o.) successfully controlled the elevated levels of COX-2 and NF-κB in DOX-induced brain tissues. Regarding TNF-α levels, only the higher dose of QUET (20 mg/kg, p.o.) resulted in considerable reduction after DOX induction. Recently, QUET was shown to reduce diabetes-induced neuroinflammation by inhibiting astrocyte and microglia activation, as well as by lowering TNF-α and monocyte chemoattractant protein-1 (MCP-1) levels in the mouse hippocampus and cerebrospinal fluid [22]. In LPS-induced neuroinflammation in mice, QUET and its metabolite norquetiapine reduced the expression of the pro-inflammatory cytokine interferon (IFN)-γ and increased the expression of the anti-inflammatory cytokine IL-10 in the hippocampus [15].

In continuation of the earlier discussion, increased peripheral TNF-α levels triggered by DOX cause microglia in the brain to become activated after crossing the BBB, which further releases TNF-α and causes oxidative stress, mitochondrial dysfunction, and cellular apoptosis, leading to cognitive decline [43]. In addition, the formation of mitochondrial calcium-mediated permeability transition pores by DOX-induced oxidative stress also contributes to mitochondrial enlargement, membrane degradation, the release of apoptotic proteins, and eventually, mitochondrial dysfunction and neurodegeneration [45,46]. The two main Bcl-2 family proteins, Bax and Bcl-2, are essential regulators of the cell death process and act to either promote or prevent cell death. Among these, the pro-apoptotic member Bax contributes to the apoptosis process by opening pores found in the outer membrane of cellular mitochondria and releasing cytochrome c, which initiates the apoptosis process. When cytochrome c is released, caspases are activated, resulting in increased cell destruction. In contrast, the anti-apoptotic protein Bcl-2 defends the pore’s opening in the mitochondrial membrane due to Bax activation and stops cell death [5,47]. This is consistent with our finding that DOX induction was evidenced by a reduction in Bcl-2 and an elevation of Bax and Caspase-3 proteins in DOX-induced brain tissues. Moreover, in addition to triggering neuronal death, Caspase family proteins regulate the structural and functional plasticity of synapses. Specifically, when caspase-3 is activated, the majority of its cellular targets are subjected to proteolytic destruction, leading to cell death [48]. Evidence supports that the elevation of DOX-induced inflammatory mediators in this study, like TNF-α and NF-κB, also mediate apoptosis regulation, particularly Caspase-3 activation, and might lead to neuronal damage and cognitive deficits [5,49,50,51]. Our results showed that QUET was capable of ameliorating the DOX-induced apoptosis process by its anti-apoptotic ability through enhancing Bcl-2 levels and protecting pro-apoptotic vulnerability by reducing Bax and Caspase-3 activities in the rat brain. Previously, chronic administration of QUET was shown to restore the phencyclidine-induced increase in Bax and decrease Bcl-XL levels in the posterior cingulate cortex area of the brain [20]. Furthermore, cerebral Bcl-2 protein expression was up-regulated by QUET in TG-AD mice [19]. Additionally, in rats with cerebral ischemia, QUET could protect against apoptosis in the penumbral region [52].

## 4. Materials and Methods

### 4.1. Animals 

Twenty-four Sprague Dawley rats (College of Pharmacy, Qassim University, Saudi Arabia) aged approximately twelve weeks (150–200 g body weight) on the day of the experiment were used. All the procedures of the present study were reviewed and permitted (Approval Number: 23-20-13) by the Committee of Research Ethics, Deanship of Scientific Research, Qassim University. The rats were grouped into four and numbered 1–6 (*n* = 6), each group by a random method. The grouped rats were housed, three per cage, and allowed free access to water and a standard pellet diet (First Milling Company, Jeddah, Saudi Arabia). Housing conditions were maintained at normal room temperature (22 ± 1 °C) with a continuous light–dark cycle from the day of acclimatization to the end of the experiment. 

### 4.2. Treatment Groups and Schedule 

**Figure 7:**
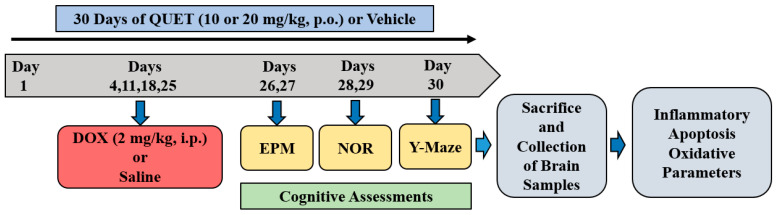
Timeline of the experiments using rats. Animals were administered with vehicle or quetiapine (QUET; 10 or 20 mg/kg, p.o.) for 30 days. To induce neuronal toxicity, four injections of doxorubicin (DOX; 2 mg/kg, i.p.; days 4, 11, 18, and 25) were given to all groups except the control group. The elevated plus-maze (EPM) assessments were conducted on day 26 (training) and day 27 (retention). On day 28 (habituation) and day 29 (training and test sessions), the novel object recognition (NOR) test was conducted. Both sessions (training and test sessions) of the Y-Maze test were conducted on day 30. After the Y-Maze test, all the animals were sacrificed, and brain tissues were collected for ELISA tests.

A total of thirty days of experimental schedule was followed for the drug administration and maze procedures (Figure 7). Animals were divided into four groups: Control (vehicle + normal saline), DOX-induced (vehicle + doxorubicin), QUET10 + DOX (quetiapine 10 mg/kg + doxorubicin), and QUET20 + DOX (quetiapine 20 mg/kg + doxorubicin). QUET (DEEF Pharmaceutical, Al Badaye, Saudi Arabia) was suspended in 0.5% *w/v* carboxymethyl cellulose (vehicle) and administered orally (p.o.) for 30 days. DOX (Fresenius Kabi Oncology Ltd., Pune, India) was diluted with normal saline (NS) and injected in four doses intraperitoneally (i.p.) on days 4, 11, 18, and 25. The doses of QUET treatment [15,20,21] and DOX-induced toxicity [5,24] were determined according to previous reports. The maze procedures were performed from day 27 to day 30 of the entire timeline cycle: day 26 (acquisition) and day 27 (retention) for EPM, day 28 (habituation) and day 29 (training and test sessions) for NOR, and day 30 (training and test sessions) for Y-Maze. At the end of the maze tests, brain tissue was collected from each rat for ELISA analysis (Figure 7). 

### 4.3. Cognitive Assessments

#### 4.3.1. Elevated Plus Maze (EPM) 

The EPM test is used to analyze the learning (acquisition) and memory (retention) capacity of animals based on their transfer latency (TL) performance [53]. In brief, it is a wooden maze with two open arms (50 cm × 10 cm) and two enclosed arms (50 cm × 10 cm × 40 cm). In the course of experiments, a height of 50 cm from the floor is maintained. Here, TL is defined as “the time taken by the animals to enter any one of the enclosed arms from a start point of the open arm” [5,24]. The EMP procedures were performed for a total of two days, on days 26 and 27 of the timeline. On day 1 of the test, the rats were allowed to explore the maze from a fixed point of anyone’s open-arm end, and the TL values were recorded in seconds. After 24 h (day 2), the procedure was repeated, and the TL values were noted again. The TLs of day 1 reflected acquisition performance, and day 2 considered the retention capacity of animals. 

#### 4.3.2. Novel Object Recognition (NOR)

The NOR test is utilized to assess the recognition memory of animals using two different objects. A wooden box (80 cm × 60 cm × 40 cm), two similar cylindrical objects considered familiar objects (FO1 and FO2), and a rectangular object considered a novel object (NO), were used for the NOR test [5]. The procedures were conducted for two days (days 28 and 29) and divided into three different phases. The first phase was habituation (day 28), when each rat was allowed to freely explore the wooden box without any objects for five minutes. After 24 h (day 29), the second phase was conducted as a training session. During this training session, each animal was allowed to explore two familiar objects (FO1 and FO2) for five minutes. The objects were fixed at two selected corners at a distance of 10 cm from the wall of the wooden box to allow rats free exploration. 4 h later, the third phase was initiated as a test session. During the test session, rats were allowed to explore one familiar object (FO1) and one novel object (NO) for five minutes. The exploration times for FO1 and NO were recorded during the test session. Exploration time is considered “the time spent by each animal when directing its nose to an object with a distance ≤2 cm or touching or sniffing the object”. The discrimination capability of animals between NO and FO was explained by calculating the percentage of discrimination index (PDI) [5].

#### 4.3.3. Y-Maze

In principle, the Y-maze allows us to examine an animal’s ability to explore a novel arm and its tendency to spend time in a new environment [5,24]. In the present test, rats were allowed to explore a wooden Y-shaped apparatus consisting of three arms with a 120° angle between each arm. Each arm was 50 cm long, 10 cm wide, and 30 cm high. To follow the procedure, two phases, namely training and test sessions, were conducted on day 30. Among the three arms, arm ‘A’ was considered the novel one, and arm ‘B’ was fixed as the starting arm. The novel arm was kept closed during the training session, and each animal was allowed to explore arms ‘B’ and ‘C’ for five minutes to familiarize themselves. After 4 h, the test session was initiated, and each animal was allowed to explore all three arms for five minutes. During the test session, the number of entries and time spent by the animals in each arm were recorded. Finally, the percentage of time spent in the novel arm by each animal was calculated according to our previous reports [5,24]. 

### 4.4. Biochemical Analysis—Enzyme-Linked Immunosorbent (ELISA) Assay

#### 4.4.1. Preparation of Brain Homogenate 

At the end of the behavioral experiments on day 30, all the rats were faintly anesthetized with diethyl ether and sacrificed via cervical decapitation. From the skull, brain tissues were isolated cautiously and added to cold phosphate-buffered saline (pH 7.6) for further homogenization. The collected brain tissues were homogenized immediately after isolation using a glass-wise stir homogenizer (Inco India, Ambala, India) and then centrifuged at 4000 rpm for ten minutes. The collected homogenates were stored in a deep freezer (−80 °C) until further use. The total protein content of each sample was analyzed using the BCA assay protocol. 

#### 4.4.2. Neuronal Oxidative Stress

To estimate oxidative parameters, oxidative (MDA) and antioxidant (GSH and CAT) markers were estimated in the present research. Rat double antibody sandwich malondialdehyde (MDA; MBS 268427) and glutathione (GSH; MBS 265966) and rat sandwich catalase (CAT; 2704433) ELISA kits (MyBioSources Inc., San Diego, CA, USA) were performed as per the manuals present in the assay kits. 

#### 4.4.3. Neuronal Inflammation 

Three selective inflammatory marker levels in brain homogenates were targeted to analyze the effect of QUET on neuroinflammation. Rat sandwich ELISA kits for cyclooxygenase-2 (COX-2; MBS 160196), nuclear factor kappa B (NF-κB; MBS 764450), and tumor necrosis factor-alpha (TNF-α; MBS824824) from MyBioSources (San Diego, CA, USA) were used according to the manufacturer’s manuals. 

#### 4.4.4. Neuronal Apoptosis

Selectively, one anti-apoptotic portion (Bcl2) and two pro-apoptotic proteins (Bax and Caspase-3) were estimated in the brain homogenate. Rat sandwich B-cell lymphoma 2 (Bcl2; MBS 452319), Bcl2 associated X protein (Bax; MBS 2703209), and rat competitive cysteinyl aspartate specific proteinase 3 (MBS 261814) ELISA kits (MyBioSources Inc., San Diego, CA, USA) were used for apoptosis analysis. The assay procedures were performed in accordance with the manual guidelines. 

### 4.5. Statistical Analysis

The mean and standard deviation were used to represent all results. GraphPad 9.5.0 version (GraphPad Software Inc., San Diego, CA, USA) was performed for one-way ANOVA analysis to determine the degree of significance levels among all groups, followed by a Tukey–Kramer post hoc test to determine the significance levels between two selected groups. In particular, to compare n the corresponding groups of FO1 and NO in the NOR test, Student’s unpaired *t*-test was used. A *p*-value ≤ 0.05 was reflected in the threshold for statistical significance.

## 5. Conclusions

Our findings concluded that thirty days of QUET administration improved the cognitive ability of DOX-induced rats (as indicated by shortened transfer latency in the elevated plus maze), improved the discrimination capability of novel objects from familiar objects in the novel object recognition test, and enhanced novel maze performance in the Y-maze test. In terms of mechanisms, QUET might reduce brain oxidative insults by altering MDA and GSH levels, reduce neuroinflammation by controlling the elevation of COX-2, NF-κB, and TNF-α levels, and reduce neuronal apoptosis by restoring normal levels of Bcl-2, Bax, and Caspase-3 proteins. Further studies need to be conducted to better understand the mechanisms by which QUET protects against DOX-induced cognitive dysfunctions in experimental animals.

## Data Availability

The data presented in this study are available from the corresponding author upon reasonable request.

## References

[1] Morean D.F., O’Dwyer L., Cherney L.R. (2015). Therapies for cognitive deficits associated with chemotherapy for breast cancer: A systematic review of objective outcomes. Arch. Phys. Med. Rehabil..

[2] Philpot R.M., Ficken M., Wecker L. (2016). Doxorubicin and cyclophosphamide lead to long-lasting impairment of spatial memory in female, but not male mice. Behav. Brain Res..

[3] Alshehri S., Assiri H., Alsalem M., Alharbi M.A. (2022). Secondary psychosis following neoadjuvant AC-T chemotherapy for triple-negative breast cancer: Case report and literature review of psychosis postchemotherapy. Case Rep. Psychiatry.

[4] Keefe R.S., Fenton W.S. (2007). How should DSM-V criteria for schizophrenia include cognitive impairment?. Schizophr. Bull..

[5] Mani V., Rabbani S.I., Shariq A., Amirthalingam P., Arfeen M. (2022). Piracetam as a therapeutic agent for doxorubicin-induced cognitive deficits by enhancing cholinergic functions and reducing neuronal inflammation, apoptosis, and oxidative stress in rats. Pharmaceuticals.

[6] Cardoso C.V., de Barros M.P., Bachi A.L.L., Bernardi M.M., Kirsten T.B., de Fátima Monteiro Martins M., Rocha P.R.D., da Silva Rodrigues P., Bondan E.F. (2020). Chemobrain in rats: Behavioral, morphological, oxidative and inflammatory effects of doxorubicin administration. Behav. Brain Res..

[7] Liao D., Shangguan D., Wu Y., Chen Y., Liu N., Tang J., Yao D., Shi Y. (2023). Curcumin protects against doxorubicin induced oxidative stress by regulating the Keap1-Nrf2-ARE and autophagy signaling pathways. Psychopharmacology.

[8] Alhowail A.H., Pinky P.D., Eggert M., Bloemer J., Woodie L.N., Buabeid M.A., Bhattacharya S., Jasper S.L., Bhattacharya D., Dhanasekaran M. (2021). Doxorubicin induces dysregulation of AMPA receptor and impairs hippocampal synaptic plasticity leading to learning and memory deficits. Heliyon.

[9] Lyu W., Ouyang M., Ma X., Han T., Pi D., Qiu S. (2021). Kai-Xin-San attenuates doxorubicin-induced cognitive impairment by reducing inflammation, oxidative stress, and neural degeneration in 4T1 breast cancer mice. Evid. Based Complement. Alternat. Med..

[10] Wang X.M., Walitt B., Saligan L., Tiwari A.F., Cheung C.W., Zhang Z.J. (2015). Chemobrain: A critical review and causal hypothesis of link between cytokines and epigenetic reprogramming associated with chemotherapy. Cytokine.

[11] Du J., Zhang A., Li J., Liu X., Wu S., Wang B., Wang Y., Jia H. (2021). Doxorubicin-induced cognitive impairment: The mechanistic insights. Front. Oncol..

[12] Ren X., Keeney J.T.R., Miriyala S., Noel T., Powell D.K., Chaiswing L., Bondada S., St Clair D.K., Butterfield D.A. (2019). The triangle of death of neurons: Oxidative damage, mitochondrial dysfunction, and loss of choline-containing biomolecules in brains of mice treated with doxorubicin. Advanced insights into mechanisms of chemotherapy induced cognitive impairment (“chemobrain”) involving TNF-α. Free Radic. Biol. Med..

[13] Sardi I., la Marca G., Cardellicchio S., Giunti L., Malvagia S., Genitori L., Massimino M., de Martino M., Giovannini M.G. (2013). Pharmacological modulation of blood-brain barrier increases permeability of doxorubicin into the rat brain. Am. J. Cancer Res..

[14] Licht T., Sasson E., Bell B., Grunewald M., Kumar S., Kreisel T., Ben-Zvi A., Keshet E. (2020). Hippocampal neural stem cells facilitate access from circulation via apical cytoplasmic processes. eLife.

[15] Jaehne E.J., Corrigan F., Toben C., Jawahar M.C., Baune B.T. (2015). The effect of the antipsychotic drug quetiapine and its metabolite norquetiapine on acute inflammation, memory and anhedonia. Pharmacol. Biochem. Behav..

[16] Riedel M., Spellmann I., Strassnig M., Douhet A., Dehning S., Opgen-Rhein M., Valdevit R., Engel R.R., Kleindienst N., Müller N. (2007). Effects of risperidone and quetiapine on cognition in patients with schizophrenia and predominantly negative symptoms. Eur. Arch. Psychiatry Clin. Neurosci..

[17] Riedel M., Schennach-Wolff R., Musil R., Dehning S., Cerovecki A., Opgen-Rhein M., Matz J., Seemüller F., Obermeier M., Engel R.R. (2010). Neurocognition and its influencing factors in the treatment of schizophrenia-effects of aripiprazole, olanzapine, quetiapine and risperidone. Hum. Psychopharmacol..

[18] He J., Luo H., Yan B., Yu Y., Wang H., Wei Z., Zhang Y., Xu H., Tempier A., Li X. (2009). Beneficial effects of quetiapine in a transgenic mouse model of Alzheimer’s disease. Neurobiol. Aging.

[19] Luo G., Liu M., He J., Guo H., Xue M., Wang X., Li X.M. (2014). Quetiapine attenuates recognition memory impairment and hippocampal oxidative stress in a transgenic mouse model of Alzheimer’s disease. Neuroreport.

[20] He J., Xu H., Yang Y., Rajakumar D., Li X., Li X.M. (2006). The effects of chronic administration of quetiapine on the phencyclidine-induced reference memory impairment and decrease of Bcl-XL/Bax ratio in the posterior cingulate cortex in rats. Behav. Brain Res..

[21] He J., Yang Y., Yu Y., Li X., Li X.M. (2006). The effects of chronic administration of quetiapine on the methamphetamine-induced recognition memory impairment and dopaminergic terminal deficit in rats. Behav. Brain Res..

[22] Wang K., Song F., Wang H., Wang J.H., Sun Y. (2019). Quetiapine attenuates the neuroinflammation and executive function deficit in streptozotocin-induced diabetic mice. Mediat. inflamm..

[23] Kciuk M., Gielecińska A., Mujwar S., Kołat D., Kałuzińska-Kołat Ż., Celik I., Kontek R. (2023). Doxorubicin-an agent with multiple mechanisms of anticancer activity. Cells.

[24] Mani V., Arfeen M., Rabbani S.I., Shariq A., Amirthalingam P. (2022). Levetiracetam ameliorates doxorubicin-induced chemobrain by enhancing cholinergic transmission and reducing neuroinflammation using an experimental rat model and molecular docking study. Molecules.

[25] Mani V., Arfeen M., Dhaked D.K., Mohammed H.A., Amirthalingam P., Elsisi H.A. (2023). Neuroprotective effect of methanolic Ajwa seed extract on lipopolysaccharide-induced memory dysfunction and neuroinflammation: In vivo, molecular docking and dynamics studies. Plants.

[26] Wang H.N., Peng Y., Tan Q.R., Chen Y.C., Zhang R.G., Qiao Y.T., Wang H.H., Liu L., Kuang F., Wang B.R. (2010). Quetiapine ameliorates anxiety-like behavior and cognitive impairments in stressed rats: Implications for the treatment of posttraumatic stress disorder. Physiol. Res..

[27] Mathiasen J.R., DiCamillo A. (2010). Novel object recognition in the rat: A facile assay for cognitive function. Curr. Protoc. Pharmacol..

[28] Lueptow L.M. (2017). Novel object recognition test for the investigation of learning and memory in mice. J. Vis. Exp..

[29] Mani V., Arfeen M., Sajid S., Almogbel Y. (2022). Aqueous Ajwa dates seeds extract improves memory impairment in type-2 diabetes mellitus rats by reducing blood glucose levels and enhancing brain cholinergic transmission. Saudi J. Biol. Sci..

[30] Kraeuter A.K., Guest P.C., Sarnyai Z. (2019). The Y-maze for assessment of spatial working and reference memory in mice. Methods Mol. Biol..

[31] Zhang Y., Zhang H., Wang L., Jiang W., Xu H., Xiao L., Bi X., Wang J., Zhu S., Zhang R. (2012). Quetiapine enhances oligodendrocyte regeneration and myelin repair after cuprizone-induced demyelination. Schizophr. Res..

[32] Tempier A., He J., Zhu S., Zhang R., Kong L., Tan Q., Luo H., Kong J., Li X.M. (2013). Quetiapine modulates conditioned anxiety and alternation behavior in Alzheimer’s transgenic mice. Curr. Alzheimer Res..

[33] Amin S.N., Gamal S.M., Esmail R.S., Aziz T.M., Rashed L.A. (2014). Cognitive effects of acute restraint stress in male albino rats and the impact of pretreatment with quetiapine versus ghrelin. J. Integr. Neurosci..

[34] Shokoohinia Y., Hosseinzadeh L., Moieni-Arya M., Mostafaie A., Mohammadi-Motlagh H.R. (2014). Osthole attenuates doxorubicin-induced apoptosis in PC12 cells through inhibition of mitochondrial dysfunction and ROS production. Biomed. Res. Int..

[35] Mounier N.M., Wahdan S.A., Gad A.M., Azab S.S. (2021). Role of inflammatory, oxidative, and ER stress signaling in the neuroprotective effect of atorvastatin against doxorubicin-induced cognitive impairment in rats. Naunyn. Schmiedebergs Arch. Pharmacol..

[36] Patel M. (2016). Targeting oxidative stress in central nervous system disorders. Trends Pharmacol. Sci..

[37] Del Rio D., Stewart A.J., Pellegrini N. (2005). A review of recent studies on malondialdehyde as toxic molecule and biological marker of oxidative stress. Nutr. Metab. Cardiovasc. Dis..

[38] Okudan N., Belviranlı M., Sezer T. (2022). Potential protective effect of coenzyme Q10 on doxorubicin-induced neurotoxicity and behavioral disturbances in rats. Neurochem. Res..

[39] Mahbub A.A., Le Maitre C.L., Haywood-Small S.L., Cross N.A., Jordan-Mahy N. (2015). Glutathione is key to the synergistic enhancement of doxorubicin and etoposide by polyphenols in leukaemia cell lines. Cell Death Dis..

[40] Sinha B.K., Mason R.P. (2015). Is metabolic activation of topoisomerase II poisons important in the mechanism of cytotoxicity?. J. Drug Metab. Toxicol..

[41] Ignácio Z.M., Réus G.Z., Abelaira H.M., de Moura A.B., de Souza T.G., Matos D., Goldim M.P., Mathias K., Garbossa L., Petronilho F. (2017). Acute and chronic treatment with quetiapine induces antidepressant-like behavior and exerts antioxidant effects in the rat brain. Metab. Brain Dis..

[42] Han J.H., Tian H.Z., Lian Y.Y., Yu Y., Lu C.B., Li X.M., Zhang R.L., Xu H. (2015). Quetiapine mitigates the ethanol-induced oxidative stress in brain tissue, but not in the liver, of the rat. Neuropsychiatr. Dis. Treat..

[43] Keeney J.T.R., Ren X., Warrier G., Noel T., Powell D.K., Brelsfoard J.M., Sultana R., Saatman K.E., Clair D.K.S., Butterfield D.A. (2018). Doxorubicin-induced elevated oxidative stress and neurochemical alterations in brain and cognitive decline: Protection by MESNA and insights into mechanisms of chemotherapy-induced cognitive impairment (“chemobrain”). Oncotarget.

[44] Tangpong J., Cole M.P., Sultana R., Joshi G., Estus S., Vore M., St Clair W., Ratanachaiyavong S., St Clair D.K., Butterfield D.A. (2006). Adriamycin-induced, TNF-alpha-mediated central nervous system toxicity. Neurobiol. Dis..

[45] Shaker F.H., El-Derany M.O., Wahdan S.A., El-Demerdash E., El-Mesallamy H.O. (2021). Berberine ameliorates doxorubicin-induced cognitive impairment (chemobrain) in rats. Life Sci..

[46] El-Agamy S.E., Abdel-Aziz A.K., Wahdan S., Esmat A., Azab S.S. (2018). Astaxanthin ameliorates doxorubicin-induced cognitive impairment (chemobrain) in experimental rat model: Impact on oxidative, inflammatory, and apoptotic machineries. Mol. Neurobiol..

[47] Westphal D., Dewson G., Czabotar P.E., Kluck R.M. (2011). Molecular biology of Bax and Bak activation and action. Biochim. Biophys. Acta.

[48] Means J.C., Gerdes B.C., Kaja S., Sumien N., Payne A.J., Stark D.A., Borden P.K., Price J.L., Koulen P. (2016). Caspase-3-dependent proteolytic cleavage of tau causes neurofibrillary tangles and results in cognitive impairment during normal aging. Neurochem Res..

[49] Alhowail A.H., Bloemer J., Majrashi M., Pinky P.D., Bhattacharya S., Yongli Z., Bhattacharya D., Eggert M., Woodie L., Buabeid M.A. (2019). Doxorubicin-induced neurotoxicity is associated with acute alterations in synaptic plasticity, apoptosis, and lipid peroxidation. Toxicol. Mech. Methods..

[50] Zhao X., Bausano B., Pike B.R., Newcomb-Fernandez J.K., Wang K.K., Shohami E., Ringger N.C., DeFord S.M., Anderson D.K., Hayes R.L. (2001). TNF-alpha stimulates caspase-3 activation and apoptotic cell death in primary septo-hippocampal cultures. J. Neurosci. Res..

[51] Lei Y., Hou F., Wu X., Yi Y., Xu F., Gong Q., Gao J. (2022). Brucine-induced neurotoxicity by targeting caspase 3: Involvement of PPARγ/NF-κB/apoptosis signaling pathway. Neurotox. Res..

[52] Yılmaz M.B., Tönge M., Emmez H., Kaymaz F., Kaymaz M. (2013). Neuroprotective effects of quetiapine on neuronal apoptosis following experimental transient focal cerebral ischemia in rats. J. Korean Neurosurg. Soc..

[53] Sharma A.C., Kulkarni S.K. (1992). Evaluation of learning and memory mechanisms employing elevated plus-maze in rats and mice. Prog. Neuropsychopharmacol. Biol. Psychiatry.

